# Exploring the Co-creation Value of Residents to Tourists From the Perspective of Place Attachment and Economic Benefits

**DOI:** 10.3389/fpsyg.2022.877365

**Published:** 2022-05-18

**Authors:** Han-Jen Niu, Mei-Jen Chen

**Affiliations:** Department of Management Science, Tamkang University, New Taipei City, Taiwan

**Keywords:** place attachment, economic benefits, environment costs, social–cultural welfare, co-creation

## Abstract

Local development enhances the economic capacity and quality of life of the residents and, in particular, attracts tourism to the area. The co-creative value of the residents and the tourists can improve the consensus of the residents on the sustainable development of the place. This study focuses on the factors influencing the co-creation of value between residents and visitors in the Tamsui area near Taipei. The research hypothesis is based on the components of local attachment, economic benefits brought by tourists, environmental costs, social and cultural welfare of the place, life satisfaction of the residents, and the value of co-creation between residents and tourists. A total of 430 questionnaires were collected through a questionnaire survey and statistical data were analyzed using a structural equation model, including descriptive statistical analysis, measurement reliability and validity verification, model fit, and structural model analysis to validate the research hypotheses. The study found that place attachment positively and significantly affects the economic benefits, environmental costs, and socio-cultural welfare of residents about tourists. Resident satisfaction is positively and significantly affected by the environmental costs from visitors and by socio-cultural welfare, but there is no significant impact from economic benefits. Finally, based on the findings of the study, practical recommendations were made for enhancing co-creation value between Tamsui residents and visitors, including enhancing residents’ feelings of place attachment and construction of local social culture and welfare. For the residents of Tamsui, unlike the local government and enterprises, need to be able to create value with tourists in order to have a friendly relationship with them and develop regional tourism in a sustainable manner.

## Introduction

The concept of local creation originated in the early days of the Japanese government. Given the aging population and the uneven development of urban areas, the rural areas in backward areas could be reactivated. Japan has entered an aging society since the early 1970s, with 7.1% of the Japanese population over 65 years old. Due to Japan’s economic and social development, Japan is facing increasingly serious population problems. The most prominent of these is the aging of the population, the declining birthrate of the population, the death rate higher than the world average, the high-density population environment, and the sharp decline in the population. To revitalize and sustainably develop urban and rural areas, not only have economic activities but also attract young people to work in their hometowns. Therefore, there are local creation practices, planned policies and strategies, and innovative planned policies and strategies. The results implemented in Japan have a significant effect. The local economy has begun to activate the population and the young population will return to their hometowns for business.

According to a United Nations report in 2015, the tourism industry is one of the largest sectors of the global economy. Especially, rural tourism development plays an important role in promoting economic growth, eradicating poverty, overcoming unemployment, improving personal welfare, promoting culture, and enhancing national image to strengthen national identity (Purnomo et al., 2020; Rustantono et al., 2020). Therefore, the tourism industry is expected to take major responsibility for local community development, including socio-cultural and economic issues (Tervo-Kankare et al., 2018). The community may not have yet provided much economic value for the tourism industry, various stakeholders are looking forward to the tourism industry to achieve community welfare benefits (Suranto et al., 2020). This has led to the goal of comprehensive and sustainable development of the community, which aims to provide renewable economic and social wealth. Researchers mentioned that the co-creation of value between tourists and local residents is seen as more.

Lengkeek (2001) refers to travel as enriching the experience of life, adding color to the world, leisure experiences, and imagination to enhance the quality of life (QoL). The development of tourism and its external links can improve the quality of life of the population, an issue that has received a lot of attention, especially in the poorer and more backward areas, as tourism development brings in people and economic income, which can make a good contribution to local development and welfare. If the views of local residents are taken into account in the development process, it is possible to achieve long-term success in tourism development and create value with tourists (Nunkoo and Ramkissoon, 2011). Moreover, Chen et al. (2020b) found that residents perceived economic impact was the most important aspect, while perceived negative impacts did not significantly affect resident satisfaction. However, there is no empirical research on the impact of economic benefits and environmental costs on the life satisfaction of tourists brought by the residents of Tamsui. Most of the tourists in Tamsui are domestic tourists, and the interaction between tourists and residents is close. In addition, the economic benefits brought by tourists can bring sustainable employment opportunities for local residents. There has no research been conducted to investigate what factors influence the value of co-creation between Tamsui residents and tourists.

The main objective of this study is to understand the factors and mechanisms that influence the value of co-creation between local residents and tourists in Tamsui. After literature research and analysis, a quantitative research model is proposed, including the attachment of residents to Tamsui, the economic benefits and costs of tourists, the satisfaction of residents’ lives due to the local construction and cultural benefits brought by tourism, and the perceived value of co-creation between residents and tourists. The research data was collected through questionnaires and the results were analyzed statistically in order to reflect the perceptions of local residents of Tamsui on the various outcomes brought by tourists and to support decision-making in the future development of local tourism.

The Tamsui District of New Taipei City is located in northern Taiwan, close to Taipei City. In the early days, Tamsui was a fishing village. Since the inconvenience of transportation, there are Taiwan cultural-historical remains, such as Oxford School, Hung Mao City, and Tamsui Chapel. Some areas still maintain the ancient rural lifestyle. However, in recent years, due to the rapid development of the city in Taiwan, some rural lands have begun to be developed. The government is also constructing new towns in Tamsui. Together with the rapid transportation build, Tamsui Old Street, Tamsui Ancient Culture, rural areas, and fishing ports have been changed. The style and features have brought some cultural and creative elements to Tamsui, attracting many tourists.

**Figure 1:**
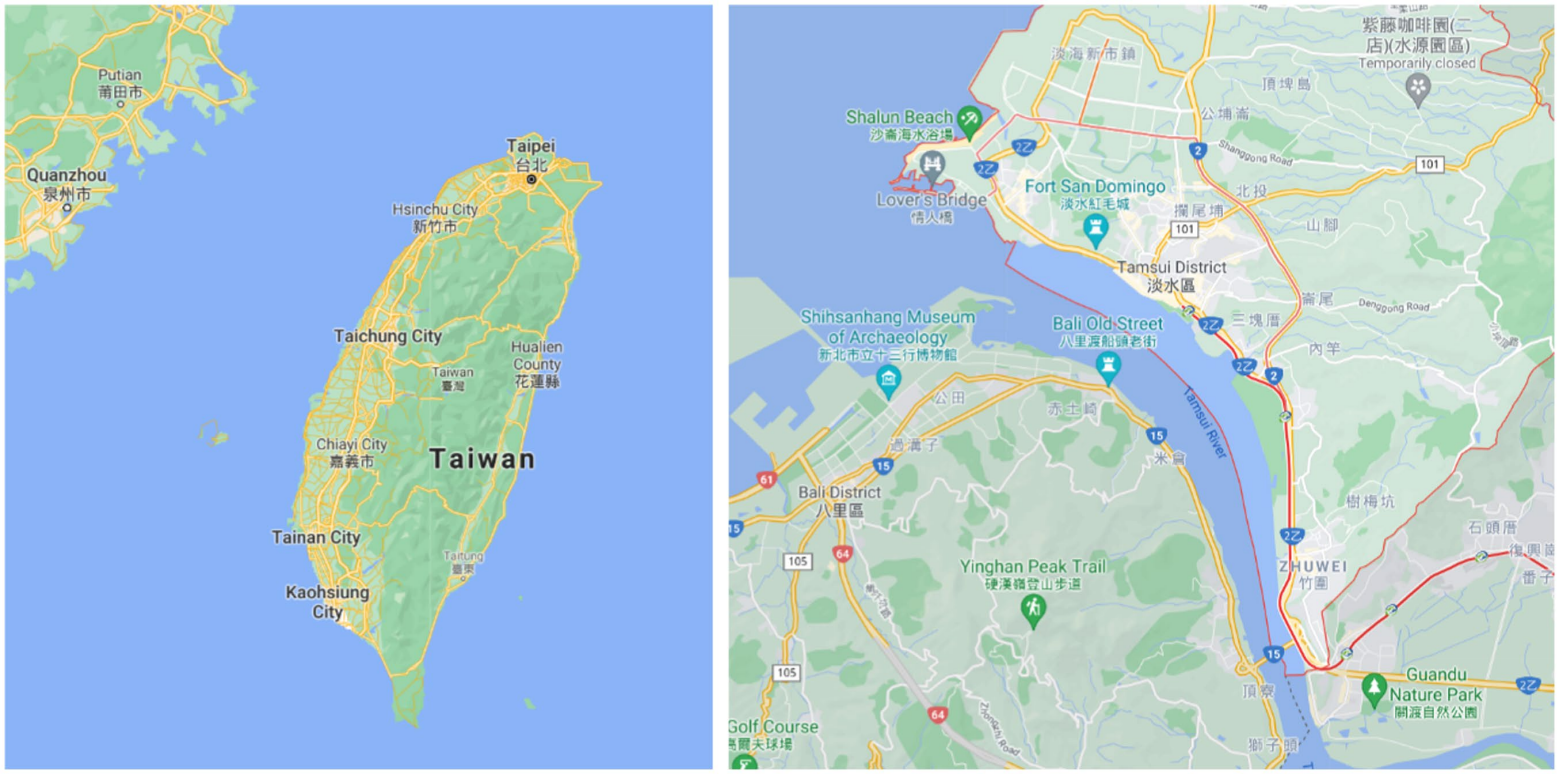
Tamsui district. Map data ©2022 Google [Google, n.d.].

Taiwan has become an aging society in 1993, which means the elderly population reached 7% of the total population. In 2017, it entered an elderly society, and the elderly population accounted for 14% of the total population (Fann and Hsu, 2010). Moreover, the population is concentrated in the cities, especially young people who are looking for jobs in the cities, which makes the development of urban and rural areas unbalanced. This situation generally occurs in rural towns across Taiwan. Since the total population of Taiwan reached its peak in 2019, population decline has begun (Tsui, 2021). The Tamsui area had a population of 173,502 at the end of 2018. It is also facing the dilemma of urban and rural development. Figure 1 shows the location of Tamsui.

## Literature Review and Hypothesis Development

### Place Attachment

Place attachment, developed from the attachment theory, aims to explore the individual’s feelings and recognition of the place in the process of place development. It is formed by the combination and mutual influence of many elements, including emotions, knowledge, beliefs about the place, etc. (Bricker and Kerstetter, 2000). Increasing competition among destinations has led to place-based attachments that have attracted widespread attention in tourism research (Shang and Luo, 2021). Place attachment emotions and functionality are divided into two parts: place identity and place dependence, including the process of place acquisition of emotional beliefs and associations. If the function of the place can meet the specific needs and behaviors of residents, it will produce functional dependence, that is, place dependence. Because of emotional attachment, people produce place attachment as the place identity (Williams et al., 1992; Daryanto and Song, 2021). Moreover, the application of the concept of place attachment can be carried out at different scales, in different industries, and with different variables (Chen et al., 2020a).

Place dependence and Place identity relate to regional development, and they have the goal of common development. Promoting residents’ participation in local activities is the driving force for local development. And this driving force will determine the overall development direction (Chang and Sung, 2010). The degree of dependence also affects the degree of local environmental management. Vaske and Kobrin (2001) found the relationship between place dependence and involvement in environmental management and environmental behavior. The stronger the degree of dependence, the more willing to be involved in the environment operation and management, or conduct environmentally responsible behaviors (Vaske and Kobrin, 2001). Moreover, the research by Chow et al. (2019) also believed that place attachment is positively correlated with satisfaction, responsible environmental behavior, and the intention to revisit the place. Based on this, place attachment is a key indicator of tourists’ environmental behavior and revisiting.

The formation of place attachment is a process, from the initial sense of place to place identity, and then the formation of place attachment (Tuan, 1977). Place identity is a symbolic or emotional attachment to the place itself. In theory, it is a complex cognitive structure. When a person is active in a specific place when positive emotions are greater than negative emotions. There will be a sense of identity with the place. Place dependence is the feelings, thoughts, and expectations of the place generated by the residents’ dependence on the function of the place. Local dependence and local identity can be linked to the common interests of the region to produce a common goal, which will promote residents to participate in local activities, will be the driving force for local development, and will determine the overall development direction and relationship. Moreover, Firestone et al. (2018) argue that place dependency would decide the residents’ support or opposition of the turbines (Firestone et al., 2018).

### Economic Benefits and Environmental Costs

In recent years, tourism can be regarded as an industry for national economic growth and local development (Brida and Risso, 2009; Tang and Tan, 2013), especially helping to increase the economic welfare of residents (Webster and Ivanov, 2014). The tourism industry is interdependent with other industries, the government, and local people. Whether it is the economy or the cost brought by tourists, everyone in the region needs to share it. The analysis of economic benefits brought by tourists can give people a better understanding of how much effect the tourism industry can bring to the region (Stynes, 1997).

Tourism activities also involve economic costs, including the direct costs of travel companies, the cost of the infrastructure provided by the government to better serve tourists, and the related costs of traffic congestion of community residents (Stynes, 1997). The community’s decision-making on tourism usually involves a debate between tourism industry supporters and residents (Tervo-Kankare et al., 2018; Gu et al., 2021). Those who hold a positive opinion are all about the economic impact (benefits) of tourism (Çalişkan and Özer, 2020), while opponents emphasize the cost of tourism.

Some Scholars believe that the development of tourism will have some adverse economic effects, such as the increase in the cost of living (Kwan and McCartney, 2005), especially the increase in real estate prices (Lorde et al., 2011). Tourism development prevails in the region, and the environment bears the brunt of negative impacts, such as pollution of the environment and air, destruction of natural resources, traffic gambling problems, and increased garbage volume, but by improving the appearance of the area, Increasing the protection of the natural environment and cultural resources, and tourism development will also have a positive impact on the environment (Ko and Stewart, 2002). In addition, due to the further development of tourism, crime rates, and traffic congestion will increase (Gursoy and Rutherford, 2004).

The value created by tourism for residents is mainly to bring economic and social and cultural benefits to the local community, while the main cost is the impact of tourism on the local environment (Nunkoo and Gursoy, 2012; Stylidis et al., 2014). In 2017, Zhibin Lin et al. expressed the residents’ perception of tourism development that the development of tourism can increase local investment and commerce, thereby providing residents with more job opportunities, increasing income, improving economic structure, etc. However, it also produces negative impacts, such as rising prices, inflation, and changes in the industrial structure (Lin et al., 2017). From the above literature, it can be found that place attachment has a positive and significant effect on the perception of economic benefits brought by tourists to residents, that is, the higher the residents’ sense of local attachment, the higher the perception of economic benefits brought by tourists. Therefore, this study proposes the following research hypothesis.

*H1:* Place attachment will positively and significantly affect residents’ perceptions of economic benefits brought by tourists.

In addition, place attachment has a positive and significant effect on the perception of environmental costs brought by tourists to local residents. The higher the perception of place attachment, the higher the perception of environmental costs brought by tourists. Therefore, this study proposes the following research hypotheses.

*H2:* Place attachment will positively and significantly affect local residents’ perceptions of environmental costs from tourists.

### Social–Culture Welfare

The investment in tourist facilities has increased the entertainment resources of residents and improved the quality of life of residents (Andereck and Nyaupane, 2011). Local residents play an important role in the sustainable development of the tourism industry. Residents’ support for the development of tourism contributes to the healthy development of tourism and successful community development (Yu et al., 2018). The development of tourism can promote cultural exchanges, revitalize local culture and provide entertainment opportunities. However, it will also increase crime opportunities for the local society, which threatens the local traditional culture (Mbaiwa, 2005).

Scholars aimed at empirical studies of tourism development on the social–culture welfare of the local community. For example, Lee et al. (2018) verified a gaming company’s corporate social responsibility would positively directly and indirectly influence residents’ perceived benefits, quality of life, and support. Moreover, Mamirkulova et al. investigated how the New Silk Road tourism infrastructure development impacting on local communities’ sustainable development and perceived quality of life. The results verified that tourism infrastructure has a direct and indirect impact on residents’ quality of life through residents’ perceived sustainable tourism development. The results would be useful for the promotion of sustainable tourism governance and residents’ welfare under the New Silk Road Infrastructural Projects (Mamirkulova et al., 2020). Furthermore, Eslami et al. said that residents’ overall satisfaction with the quality of life would affect support for sustainable tourism development. The perceptual influence of tourism on social culture has an important relationship with the non-material life field. The perceivable impact of tourism on the economy would impact material and non-material life spheres (Eslami et al., 2019).

From the above literature, in addition to the economic benefits and environmental costs that tourists bring to a place, the local government’s efforts to revitalize tourism bring better socio-cultural and welfare benefits to local residents. The higher the perception of place attachment, the higher the perception of socio-cultural and welfare benefits that the government brings to tourism. Therefore, the following hypothesis is proposed for this study.

*H3:* Place attachment will positively and significantly affect local residents’ perceptions of social–cultural welfare provided by the government for tourism development.

### Life Satisfaction

Life satisfaction can be defined as an individual’s judgment of life in many aspects (Diener, 1984; Diener and Tay, 2017), and is the most extensive structure for evaluating subjective wellbeing (Sachs et al., 2018). Satisfaction has a wide range of applications and is a very useful behavior measurement indicator, which is commonly used to measure people’s views on products, services, etc. Satisfaction is the psychological state of an individual after experiencing activities. It is affected by social factors, or external factors, such as the atmosphere and group interaction at the time (Baker and Crompton, 2000).

On the other hand, travel is a time when travelers leave home to interact with other people in other places, and the process of interaction between them affects their satisfaction with life, sense of wellbeing, and expectations for the future (Bimonte and Punzo, 2011, 2016). Travelers and residents may have different interests and expectations, so there may be mutual benefits as well as some potential conflicts between them (Lin et al., 2017). Furthermore, social exchange theory from the perspective of tourism development suggests that residents’ evaluation of the results of community tourism development affects their sense of wellbeing and support for tourism development (Andereck et al., 2005). Many studies have found that residents are more likely to support tourism and participate in tourism activities when they perceive the benefit–cost ratio to be positive (Gursoy and Rutherford, 2004). Residents’ attitudes toward tourism, their level of support for tourism development, and their perceived quality of life vary depending on the nature of the assessment. As a result, the academic tourism development literature is replete with studies examining predictors of travel attitudes, community attachment, community life satisfaction, and quality of life through indicators of economic gain, personal growth (e.g., employment), and length of stay (Woo et al., 2015).

However, there are costs associated with tourists that need to be taken into account, such as the impact of tourists on traffic, security, noise, and quality of life. Any viable tourism development needs to balance the costs and benefits (Deery et al., 2012). If a balance is not achieved, local communities may show indifference or hostility toward tourists and tourism development (Bimonte et al., 2019).

Based on the above literature, residents’ perception of the economic benefits brought by tourists affects residents’ satisfaction with their lives, and the higher the perceived economic benefits brought by tourists, the higher their satisfaction with their lives will be.

*H4:* The economic benefits brought by tourists will positively and significantly affect the life satisfaction of local residents.

*H5:* The environmental cost of tourists will negatively and significantly affect the life satisfaction of local residents.

*H6:* The social–cultural welfare will positively and significantly affect the satisfaction of local residents.

### Resident-Tourist Value Co-creation

The tourism industry to enhance regional economic development has become an important way of regional development. The economic development brought about by tourists can increase local employment opportunities, taxation, economic development, and other important effects (Kim et al., 2013). Bimonte and Punzo (2016) proposed that tourists and residents should play an equally important role in the development of the tourism industry. Many studies pay too much attention to residents and ignore tourists. In recent years, many tourism connotations have emphasized the interaction between tourists and residents. The encounter and interactive experience between tourists and residents may affect the satisfaction, happiness, and future behavior of both parties (Sharpley, 2014) Because both parties have their own interests and expectations, there are both mutual benefits and potential conflicts between the two parties (Bimonte and Punzo, 2016). Lin et al. (2017) had verified the residents’ perceived benefits and costs of tourism of the development of co-creation behaviors with tourism value.

Second, this research has improved our understanding of life satisfaction. The antecedents of residents and tourists create value together (Lin et al., 2017). This contribution is important because most studies treat life satisfaction as an outcome variable and ignore it as an influencing factor of co-creation. Moreover, a study by Woo et al. (2015) verified that residents are more likely to support tourism development and participate in tourism activities when they perceive that tourists bring a positive benefit–cost ratio. Therefore, this study proposes the research hypothesis that the higher the satisfaction level of Tamsui residents with their own lives, the higher the perceived value of co-creation with tourists.

*H7:* The life satisfaction of Tamsui residents will positively and significantly influence the value of co-creation with tourists.

In this study, the operational definitions of independent variables and dependent variables and the references to the literature are organized in Table 1.

**Table 1:** Operational definitions of research variables.

Research variables	Operational definitions	References
Place identity	I want to live in a safe, memorable, cultural, friendly, special and unique place.	Williams et al., 1992; Jorgensen and Stedman, 2001
Place dependency	This place feels like my home, it feels like I belong, it suits me very well, and it feels intimate.	
Economic benefits	Visits from tourists can improve my standard of living, increase job opportunities, improve infrastructure, and increase income.	Nunkoo and Ramkissoon, 2010; Lin et al., 2017
Environment costs	Tourists will bring noise, environmental pollution, and traffic congestion.	Nunkoo and Ramkissoon, 2010; Lin et al., 2017
Social–cultural welfare	The community has developed into a multicultural one, with more public spaces for people to interact with each other while preserving traditional culture.	Dyer et al., 2007
Life satisfaction	My life is in line with my ideal state and I live in a great state.	Diener et al., 1985
Resident–tourist value co-creation	I have a high respect for visitors and will provide them with useful information about our way of life, traditions, culture and history.	Lin et al., 2017

**Figure 2:**
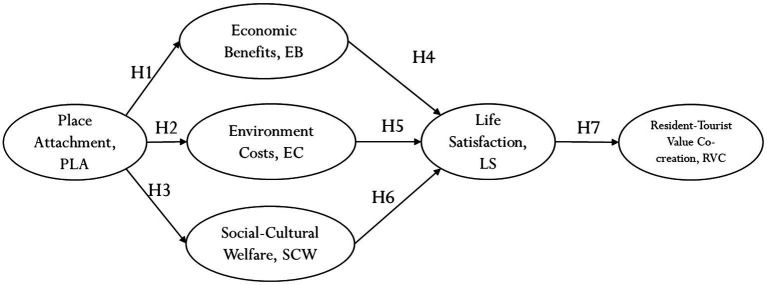
Research model.

This study proposes the following research model as shown in Figure 2.

## Methodology

### Research Objects and Questionnaire Survey

This study is aimed at residents in Tamsui, Taiwan, and explores the local attachment, life satisfaction, and the co-creation value of residents and tourists under the development of local tourism. The population of Tamsui is 173,502 at the end of 2018. This study uses online software to make questionnaires and then uses social software to promote the questionnaires. The collection time is from 2021/4/10 to 2021/5/30, and the number of questionnaires collected is 430. According to the sample size requirement formula from the survey system website (The Survey System, 2022), when the statistical confidence was 95% and the confidence interval was 5%, the sample size of the total population of Tamsui District was 384 copies. Therefore, the sample size of this research was sufficient.

### Measurement Instrument

This research questionnaire consists of two parts. One is demographic variables, such as gender, marriage, age, education, time living in this community, occupation, and monthly income. Then, in the second part of the questionnaire, the six latent variables were measured by a Likert seven-point scale ranging from “strongly disagree” (1) to “strongly agree” (7). This study would measure the research constructs, including the degree of place attachment, tourism development perception economic benefit, social and cultural welfare, environment cost, life satisfaction, and residents and tourist co-creation value. The scales and references for this study are shown in Table 2.

**Table 2:** Questionnaire items and sources.

Variable	Items	References
Place identity	I have found the life I want to live in Tamsui It makes me feel safe in Tamsui and is the best place for my life. In Tamsui is full of memories, I agree that Tamsui is more important than other places For me, living in Tamsui has a special meaning that cannot be replaced by other places I understand the local cultural background of Tamsui If Tamsui needs my help, I will try to help. I am willing to take on local public affairs duties	Jorgensen and Stedman, 2001; Williams et al., 1992
Place dependency	Tamsui makes me feel “home,” and I feel sad if I leave. Tamsui makes me feel like I belong, I feel like I’m part of this place. I cannot think of any other place I’d rather be than where I live now. There are many activities in my life that are closely related to Tamsui The experience of living in Tamsui makes me not want to leave here	
Economic benefits	The development of tourism in Tamsui will improve the standard of living Tourism development in Tamsui can create job opportunities Tourism development in Tamsui can improve infrastructure Tourism development in Tamsui can increase economic income	Nunkoo and Ramkissoon, 2010; Lin et al., 2017
Environment costs	Tourism development will create crowdedness Tourism development will cause traffic congestion Tourism development will increase noise Tourism development will increase environmental pollution	Nunkoo and Ramkissoon, 2010; Lin et al., 2017
Social–cultural welfare	Tourism development will provide more parks and recreational facilities The development of tourism will promote local cultural activities Tourism can enhance cultural exchange Tourism can contribute to the preservation of local culture	Dyer et al., 2007
Life satisfaction	My life is mostly close to my ideal My living condition is quite good I am very satisfied with my life now I have gotten the important things I want in my life (health, money, etc.) If I could start my life over, I would hardly change anything	Diener et al., 1985
Resident–tourist value co-creation	I treated tourists with high esteem I provided tourists with useful information, such as transport, attractions, restaurants, hotel, and others I provided tourists with information on our way of life, traditional culture, and history	Lin et al., 2017

### Data Analysis

The data analysis of this study will be carried out in several stages: descriptive statistical analysis, measurement model confirmation, and structural equation model verification. Among them, based on the statistics of the background data, sample statistical data would be measured, including the number and percentage. In addition, the average and standard deviation of the response scores of each variable would be calculated. Then, a two-step analysis method is used to execute the measurement model and the structural model. In this study, AMOS 21.0 was used to perform statistical analysis. The first is to use Confirmatory Factor Analysis (CFA) to test the reliability and validity of the items, which includes measuring the degree of internal consistency of each variable, as well as convergence and differentiation validity. Next, Structural Equation Modeling (SEM) would be used for analysis to test the fit of the research model. Then, this study would verify the hypotheses of the research framework, including path analysis and mediating effect analysis.

## Results

### Demographic Variables

The distribution of the sample data in this study includes: 231 (53.7%) are male; 199 (46.3%) are aged 31–50; 242 (27.4%) are college/university educated; 293 (68.1%) are married; 137 (31.9%) have lived in the community for more than 21 years; 41.9% have disposable monthly income of 20,000–50,000 dollars; 350 (81.4%) are living in their own home, as shown in Table 3.

**Table 3:** Demographic variables.

Category	Label	Frequency	Percent	Category	Label	Frequency	Percent
Gender	Female	199	46.3	Marital status	Married	293	68.1
	Male	231	53.7		Unmarried	137	31.9
Age (year)	Under 20 years old	6	1.4	How long live in the community (year)	Under 3 years	66	15.3
	21–30 years old	45	10.5		3–5 years	65	15.1
	31–50 years old	199	46.3		6–10 years	71	16.5
	51–60 years old	98	22.8		11–20 years	91	21.2
	Over 61 years old	82	19.0		Over 21 years	137	31.9
Education level	Junior high school	9	2.1	Disposable monthly income (NT $)	Under 20,000	50	11.6
	High school	61	14.2		20,000–50,000	180	41.9
	college/university educated	242	56.3		50,000–100,000	128	29.8
	Graduate school	118	27.4		Over 100,000	72	16.7
Own/leasing house	Own house	350	81.4				
	Leasing house	80	18.6				

### Descriptive Statistics

In this study, the score of items’ means was between 4.095 and 5.272, standard deviations were between 1.201–1.425, Skewness values were between −0.533 and 0.222, and Kurtosis values were between −0.844 and 0.279. All of them meet Kline (2015) criteria of the absolute value of Skewness less than 2 and the absolute value of Kurtosis less than 7. If they meet these two criteria, they are said to have a normal distribution. Therefore, the results of this study are consistent with the normative distribution, as shown in Table 4.

**Table 4:** Descriptive statistics.

Item	*N*	Mean	Std. deviation	Skewness	Std. error of skewness	Kurtosis	Std. error of kurtosis
PI1	430	4.867	1.313	−0.238	0.118	−0.065	0.235
PI2	430	4.914	1.319	−0.276	0.118	−0.159	0.235
PI3	430	4.774	1.387	−0.291	0.118	−0.233	0.235
PI4	430	4.658	1.428	−0.261	0.118	−0.302	0.235
PI5	430	4.635	1.386	−0.234	0.118	−0.316	0.235
PI6	430	4.891	1.347	−0.076	0.118	−0.591	0.235
PI7	430	4.584	1.323	0.222	0.118	−0.648	0.235
PD1	430	4.802	1.432	−0.290	0.118	−0.262	0.235
PD2	430	4.888	1.364	−0.317	0.118	−0.196	0.235
PD3	430	4.326	1.544	−0.224	0.118	−0.426	0.235
PD4	430	4.742	1.361	−0.328	0.118	0.021	0.235
PD5	430	4.623	1.475	−0.256	0.118	−0.449	0.235
EB1	430	4.677	1.460	−0.389	0.118	−0.015	0.235
EB2	430	4.879	1.377	−0.464	0.118	0.279	0.235
EB3	430	4.912	1.397	−0.440	0.118	0.064	0.235
EB4	430	4.953	1.412	−0.397	0.118	−0.015	0.235
SCW1	430	4.958	1.454	−0.343	0.118	−0.436	0.235
SCW2	430	5.142	1.328	−0.280	0.118	−0.353	0.235
SCW3	430	5.235	1.302	−0.264	0.118	−0.515	0.235
SCW4	430	5.016	1.431	−0.364	0.118	−0.334	0.235
EC1	430	5.216	1.201	−0.180	0.118	−0.346	0.235
EC2	430	5.272	1.207	−0.288	0.118	−0.109	0.235
EC3	430	5.193	1.278	−0.332	0.118	−0.315	0.235
EC4	430	5.195	1.341	−0.465	0.118	−0.091	0.235
RV1	430	5.200	1.284	−0.391	0.118	−0.161	0.235
RV2	430	5.219	1.339	−0.533	0.118	0.129	0.235
RV3	430	5.205	1.288	−0.347	0.118	−0.287	0.235
LS1	430	4.616	1.318	−0.291	0.118	−0.276	0.235
LS2	430	4.721	1.330	−0.302	0.118	−0.226	0.235
LS3	430	4.714	1.353	−0.388	0.118	−0.038	0.235
LS4	430	4.451	1.434	−0.202	0.118	−0.536	0.235
LS5	430	4.095	1.645	−0.071	0.118	−0.844	0.235

### Convergent Validity

The measurement model was estimated by the Maximum Likelihood Estimation, and the estimated parameters included factor loadings, reliability, convergent validity, and discriminant validity. Table 5 provides standardized factor loadings, composite reliability, Cronbach’s alpha and average variance extracted values. Fornell and Larcker (1981) suggested that the standardized factor loading for each item should be higher than. 50, the composite reliability and Cronbach’s alpha value should be higher than 0.60, and the average variance extracted should be higher than 0.50. As shown in Table 5, the standardized factor loadings ranged from 0.638 to 0.929, which are all within a reasonable range. The reliability of the study constructs ranged from 0.772 to 0.950, with all exceeding 0.7. Cronbach’s alpha values of constructs were from 0.804 to 0.946. The average variance extractions ranged from 0.628 to 0.791, all above 0.5, which is within the criteria. The measurement model in this study meets the recommended criteria.

**Table 5:** Convergent validity.

Construct	Item	Std.	CR	Cronbach’s alpha	AVE
Place dependence	PD1	0.904	0.936	0.935	0.745
	PD2	0.911			
	PD3	0.829			
	PD4	0.798			
	PD5	0.867			
Place identity	PI1	0.847	0.917	0.917	0.613
	PI2	0.864			
	PI3	0.797			
	PI4	0.805			
	PI5	0.749			
	PI6	0.759			
	PI7	0.638			
Economic benefits	EB1	0.827	0.944	0.943	0.809
	EB2	0.928			
	EB3	0.923			
	EB4	0.916			
Social–cultural welfare	SCW1	0.878	0.942	0.941	0.804
	SCW2	0.916			
	SCW3	0.910			
	SCW4	0.881			
Environment cost	EC1	0.878	0.939	0.938	0.795
	EC2	0.913			
	EC3	0.915			
	EC4	0.859			
Resident–tourist value co-creation	RV1	0.895	0.936	0.936	0.830
	RV2	0.927			
	RV3	0.911			
Life satisfaction	LS1	0.900	0.950	0.946	0.791
	LS2	0.929			
	LS3	0.920			
	LS4	0.891			
	LS5	0.800			
Place attachment	Place identity	0.792	0.772	0.804	0.628
	Place dependence	0.793			

Std, standardized factor loadings; CR, composite reliability; AVE, average variance extracted.

### Discriminant Validity

Fornell and Larcker (1981) suggested that discriminant validity should also consider the relationship between convergent validity and construct correlation. Therefore, they suggested that the square root of AVE for each construct should be greater than the correlation coefficient between the constructs, which indicated the discriminant validity of the model. As shown in Table 6, the root square of AVE for each diagonal construct of this study is greater than the off-diagonal correlation coefficients.

**Table 6:** Discriminant validity.

	AVE	EB	SCW	EC	RVC	LS	PLA
EB	0.809	0.899					
SCW	0.804	0.730	0.897				
EC	0.795	0.367	0.485	0.892			
RVC	0.830	0.668	0.756	0.558	0.911		
LS	0.791	0.374	0.487	0.371	0.532	0.889	
PLA	0.628	0.544	0.565	0.541	0.594	0.756	0.792

EB, Economic benefits; SCW, Social-cultural welfare; EC, Environment cost; RVC, Resident-tourist value co-creation; LS, life Satisfaction; PLA, Place attachment.

### Model Fit

Since the structural equation model sample is larger than 200, the model fit would be not good due to the large chi-square value. The model fit needs to be corrected by the Bootstrap method (Bollen and Stine, 1992). The results of the Bollen–Stine Bootstrap correction model are shown in Table 7. After the Bollen–Stine Bootstrap correction, the fit indicators of this study passed, Root Mean Square Error of Approximation (RMSEA)0.032 < 0.08; Non-Normed Fit Index (NNFI) 0.985 > 0.9; Normed Fit Index (NFI)0.956 > 0.9; Comparative Fit Index (CFI)0.986 > 0.9; Goodness of Fit (GFI)0.956 > 0.9; Adjust Goodness of Fit (AGFI)0.947 > 0.9 indicating that the results of this study are acceptable.

**Table 7:** Model fit.

Model fit	Criteria	Model fit of the research model
*χ* ^2^	The small the better	649.334
DF	The large the better	455
Normed Chi-sqr (*χ*^2^/DF)	1 < *χ*^2^/DF < 3	1.427
RMSEA	<0.08	0.032
TLI (NNFI)	>0.9	0.985
NFI	>0.9	0.956
CFI	>0.9	0.986
GFI	>0.9	0.956
AGFI	>0.9	0.947

RMSEA, Root Mean Square Error of Approximation; NNFI, Non-Normed Fit Index; NFI, Normed Fit Index; CFI, Comparative Fit Index; GFI, Goodness of Fit; AGFI, Adjust Goodness of Fit.

### Path Analysis

The results of the path coefficients can be seen in Table 8. First, place attachment significantly impacts the economic benefits of tourism (*b* = 0.964, *p* < 0.001). Then, place attachment significantly influences the social–cultural benefits of tourism (*b* = 1.084, *p* < 0.001). Then, place attachment impacts perceived costs of tourism significantly (*b* = 0.732, *p* < 0.001). Moreover, the economic benefits of tourism do not have a significant impact on life satisfaction (*b* = 0.054, *p* = 0.295 > 0.05). Then, the social–cultural benefits of tourism influence life satisfaction significantly (*b* = 0.370, *p* < 0.001). Furthermore, perceived costs of tourism impact life satisfaction significantly (*b* = 0.206, *p* < 0.001). Finally, life satisfaction significantly impacts resident–tourist value co-creation (*b* = 0.531, *p* < 0.001).

**Table 8:** Regression coefficient.

Hypothesis	DV	IV	Unstd.	SE	Unstd./SE	*p*-value	Std.	*R* ^2^	Result
H1	EB	PLA	0.964	0.085	11.310	0.000	0.691	0.477	Supported
H2	EC	PLA	0.732	0.071	10.321	0.000	0.602	0.362	Supported
H3	SCW	PLA	1.084	0.089	12.176	0.000	0.742	0.551	Supported
H4	LS	EB	0.054	0.052	1.046	0.295	0.056	0.293	Not supported
H5		EC	0.206	0.057	3.607	0.000	0.185		Supported
H6		SCW	0.370	0.052	7.122	0.000	0.398		Supported
H7	RVC	LS	0.531	0.045	11.679	0.000	0.548	0.300	Supported

DV, Dependent Variable; IV, Independent Variable; Unstd., Unstandardized Regression Coefficient; SE, Standard Error; Std., Standardized Regression Coefficient; EB, Economic benefits; SCW, Social–cultural welfare; EC, Environment cost; RVC, Resident–tourist value co-creation; LS, Life Satisfaction; PLA, Place attachment.

The purpose of the study hypothesis is to understand the significance of the independent variables to the dependent variables in the study model. A *R*^2^ value greater than 0.670 indicates good explanatory power, *R*^2^ between 0.330 and 0.670 indicates acceptable explanatory power, and *R*^2^ less than 0.190 indicates poor explanatory power (Chin, 1998). In this study, the explanatory power of variance (*R*^2^) of Economic benefits (EB) is 0.477, Environment cost (EC) is 0.362, Social cultural welfare (SCW) variance (*R*^2^) is 0.551, life satisfaction (LS) variance (*R*^2^) is 0.293, resident. The explanatory power of tourist value co-creation (RVC) variance (*R*^2^) is 0.548, which indicates that the explanatory power of this study model is acceptable.

**Figure 3:**
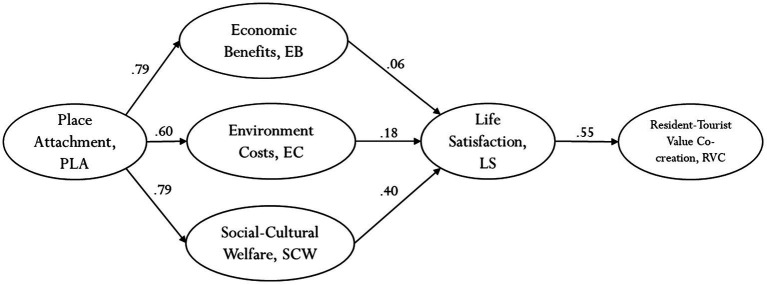
Statistical model analysis.

Figure 3 displays the statistical results.

### Analysis of Mediation Effects

Because bootstrapping mediation analysis can provide confidential intervals to examine the indirect effects, it is better than the other mediation testing methods. One of the preferable bootstrapping mediation analysis methods is bias-corrected bootstrapping (Williams and MacKinnon, 2008).

The study verified that “Bootstrap Results for Indirect Effects” from the output window and the 95% confidence interval [CI of PLA → EC → LS = (0.052–0.278); CI of PLA → SCW → LS =  (0.192–0.870)]. If the confidence interval does not include 0, the indirect effect *a* * *b* is significant and mediation is established as Table 9. Both indirect effects are valid.

**Table 9:** The analysis of indirect effects.

Effect	Point estimate	Product of coefficients			Bootstrap 1,000 times	
					Bias-corrected 95%	
		SE	*Z*-value	*p*-value	Lower bound	Upper bound
*IV to Mediator (a path)*						
PLA → EC	0.732	0.113	6.478	0.002	0.534	0.968
*Direct effects of mediator on DV (b path)*						
EC → LS	0.206	0.072	2.901	0.002	0.077	0.355
Indirect effects						
PLA → EC → LS	0.151	0.055	2.745	0.002	0.052	0.278
*IV to Mediator (a path)*						
PLA → SCW	1.084	0.283	3.830	0.001	0.753	1.835
*Direct effects of mediator on DV (b path)*						
SCW → LS	0.370	0.092	4.022	0.002	0.534	0.968
*Indirect effects*						
PLA → SCW → LS	0.401	0.171	2.345	0.002	0.192	0.870

IV, Independent Variable; DV, Dependent Variable; EB, Economic benefits of tourism; SCW, Social–cultural benefits of tourism; EC, Perceived costs of tourism; RVC, Resident-tourist value co-creation; LS, life Satisfaction; PLA, Place attachment.

## Conclusion and Discussion

The main objective of this study is to understand the value of co-creation between the residents of Tamsui and tourists through a questionnaire survey and to explore the influencing factors, including the two sub-structures of local attachment, local identity and place dependency, the economic benefits, and the environmental costs brought by tourists, the construction of local social–culture and welfare due to tourists, and the satisfaction of the residents of Tamsui. The findings of the study are as follows.

### Theoretical Contributions

#### Local Attachment Significantly Affects the Economic Benefits, Environmental Costs, and Social–Culture and Welfare of Tourists

The impact of residents on economic benefits, environmental costs, and social–culture welfare of tourists is significantly influenced by residents’ attachment to place. H1, H2, and H3 are supported. The results of this study are similar to those of Nunkoo and Gursoy (2012) and Stylidis et al. (2014). Moreover, the research results verified as the results of Alrwajfah et al. (2019). The local attachment has a clear impact on the results that tourists bring to a place, which means that the higher the degree of local attachment, the more the residents perceive the impact of tourists, with the residents being the most sensitive to the social and cultural construction of the place and its welfare. The social and cultural development of the local community is a long-term development that has a direct impact on the convenience and quality of life of the residents.

#### The Impact of Tourists on Residents’ Satisfaction With Their Lives

This study verified the effects of economic benefits, environmental costs, and local social culture and welfare brought by tourists on the satisfaction of residents in Tamsui areas. The economic benefits of tourists have no significant impact on the life satisfaction of residents, while the environmental costs of tourists and the social–culture welfare have a significant impact on the local community. The results of this study are different from those of Gursoy and Rutherford (2004) and Woo et al. (2015). The residents of Tamsui do not feel that the economic benefits brought by tourists have a positive impact on their satisfaction with life. Tourists may come to Tamsui to spend money on restaurants or heritage sites, which have no real impact on most residents, but the environmental costs brought by tourists are felt to have an impact on their satisfaction with life.

#### The Impact of Residents’ Life Satisfaction on Resident-Tourist Value Co-creation

This study verifies that the life satisfaction of Tamsui residents positively and significantly affects the co-creation value of resident tourists. In addition to significant direct effects, residents’ perceptions of place attachment indirectly influence the value of co-creation by resident tourists through economic costs and socio-cultural welfare. The results of this study were similar to those of Lin et al. (2017) and Yang et al. (2020). Tamsui is located in the suburbs of Taipei City, and most of the visitors to Tamsui are domestic tourists. Tamsui is a traditional and famous scenic spot for the people of Taiwan, and many tourists visit Tamsui many times. In recent years, there are many public landscape and leisure facilities in Tamsui, where tourists and local residents can mix and mingle and interact smoothly. The residents of Tamsui have demonstrated that the facilities built for tourists have increased their life satisfaction and positively influenced the value of co-creation with tourists.

### Management Implications

Based on the results of the study, this study proposes substantive approaches for businesses and local governments in the Tamsui area to enhance the co-creation value of residents and tourists. The recommendations are as follows.

#### Enhancing Residents’ Feelings of Place Attachment

This study found that feelings of place attachment have a significant positive impact on the economic benefits, environmental costs, and local socio-cultural wellbeing brought by tourists. Therefore, enhancing the residents’ sense of attachment to the place will help to improve the perception of the benefits of the place to visitors. Local governments can organize a variety of arts and cultural activities, especially in the form of exploring local stories and introducing people and events related to local development, in order to enhance the connection and emotion among residents. Tamsui is a place of great character, due to its geographical location and its long history. In addition to preserving the historic buildings and streets, the local government could organize local cultural events, such as street parades that incorporate early folk rituals or epidemics and incorporate modern methods of promotion and management.

#### Construction of Local Social Culture and Welfare

According to the results of this study, among the benefits and disadvantages brought by tourists, the residents of Tamsui area perceive the local social and cultural welfare construction most, such as the construction of traffic, park streetlights, iconic buildings, and other hardware construction. Tamsui area has invested in the preservation, maintenance, and promotion of local traditional folk culture. Although it was built to attract tourists, the residents feel it the most. The most concrete feeling that residents have about the value of co-creation by tourists is that there is a place to build because of their arrival. In short, local governments should build or continuously maintain public facilities while attracting tourists, so that residents will feel the value of co-creation that tourists can bring, and their acceptance of tourists will increase.

### Research Limitations and Future Research Directions

This study examines the factors that influence the co-creation of value between local residents and tourists in Tamsui. Through the collection of data from questionnaires, the research hypotheses were statistically tested to confirm the relationship between residents’ feelings of local attachment to the economic benefits, environmental costs, and local culture and social welfare brought by tourists. The study only focused on residents of the Tamsui area, the sample size was limited and no survey had been conducted on tourists. In addition, this study only investigates specific constructs. Future research could expand the scope and target population of the survey to collect more empirical data. Moreover, more dimensions, such as nostalgia and destination attractiveness, could be added to increase the tourist factors of visiting Tamsui places from the perspective of tourists, which could be used to improve the decision support of tourism development.

## Data Availability Statement

The raw data supporting the conclusions of this article will be made available by the authors, without undue reservation.

## Author Contributions

The conceptualization of this research is provided by H-JN. The original draft preparation, review and editing, and visualization are all completed by M-JC. All authors contributed to the article and approved the submitted version.

## Conflict of Interest

The authors declare that the research was conducted in the absence of any commercial or financial relationships that could be construed as a potential conflict of interest.

## Publisher’s Note

All claims expressed in this article are solely those of the authors and do not necessarily represent those of their affiliated organizations, or those of the publisher, the editors and the reviewers. Any product that may be evaluated in this article, or claim that may be made by its manufacturer, is not guaranteed or endorsed by the publisher.
